# Development and validation of a nomogram to predict cancer-specific survival in nonsurgically treated elderly patients with prostate cancer

**DOI:** 10.1038/s41598-023-44911-z

**Published:** 2023-10-18

**Authors:** Zhaoxia Zhang, Qian Cai, Jinkui Wang, Zhigang Yao, Fengming Ji, Yu Hang, Jing Ma, Hongchao Jiang, Bing Yan, Chenghao Zhanghuang

**Affiliations:** 1 Department of Urology, Kunming Children’s Hospital (Children’s Hospital affiliated to Kunming Medical University), 288 Qianxing Road, Kunming, 650228 Yunnan China; 2 Department of Urology, Chongqing Key Laboratory of Children Urogenital Development and Tissue Engineering, Chongqing Key Laboratory of Pediatrics, Ministry of Education Key Laboratory of Child Development and Disorders, National Clinical Research Center for Child Health and Disorders, China International Science and Technology Cooperation Base of Child Development and Critical Disorders, Children’s Hospital of Chongqing Medical University, Chongqing Higher Institution Engineering Research Center of Children’s Medical Big Data Intelligent Application, Chongqing, People’s Republic of China; 3 Department of Urology, Affiliated Hospital of Yunnan University (The Second People’s Hospital of Yunnan Province, Ophthalmic Hospital of Yunnan Province), Kunming, Yunnan People’s Republic of China; 4 Yunnan Key Laboratory of Children’s Major Disease Research, Kunming Children’s Hospital (Children’s Hospital Affiliated to Kunming Medical University), Yunnan Province Clinical Research Center for Children’s Health and Disease, Kunming, People’s Republic of China; 5 Science and Education Department, Kunming Children’s Hospital (Children’s Hospital affiliated to Kunming Medical University), Kunming, People’s Republic of China

**Keywords:** Cancer therapy, Urological cancer

## Abstract

Prostate Cancer (PC) is the most common male nonskin tumour in the world, and most diagnosed patients are over 65 years old. The main treatment for PC includes surgical treatment and nonsurgical treatment. Currently, for nonsurgically treated elderly patients, few studies have evaluated their prognostic factors. Our aim was to construct a nomogram that could predict cancer-specific survival (CSS) in nonsurgically treated elderly PC patients to assess their prognosis-related independent risk factors. Patient information was obtained from the Surveillance, Epidemiology and End Results (SEER) database, and our target population was nonsurgically treated PC patients who were over 65 years old. Independent risk factors were determined using both univariate and multivariate Cox regression models. A nomogram was built using a multivariate Cox regression model. The accuracy and discrimination of the prediction model were tested using the consistency index (C-index), the area under the subject operating characteristic curve (AUC), and the calibration curve. Decision curve analysis (DCA) was used to examine the potential clinical value of this model. A total of 87,831 elderly PC patients with nonsurgical treatment in 2010–2018 were included in the study and were randomly assigned to the training set (N = 61,595) and the validation set (N = 26,236). Univariate and multivariate Cox regression model analyses showed that age, race, marital status, TNM stage, chemotherapy, radiotherapy modality, PSA and GS were independent risk factors for predicting CSS in nonsurgically treated elderly PC patients. The C-index of the training set and the validation set was 0.894 (95% CI 0.888–0.900) and 0.897 (95% CI 0.887–0.907), respectively, indicating the good discrimination ability of the nomogram. The AUC and the calibration curves also show good accuracy and discriminability. We developed a new nomogram to predict CSS in elderly PC patients with nonsurgical treatment. The model is internally validated with good accuracy and reliability, as well as potential clinical value, and can be used for clinical aid in decision-making.

## Introduction

Since the U. S. Preventive Services Task Force (USPSTF) recommended using serum prostate specific antigen (PSA) for prostate cancer (PC) screening^1^. PSA screening has led to a significant increase in the diagnosis of low- and intermediate-risk prostate cancer worldwide. At present, the incidence of PC ranks first among nonskin tumours in males^2,3^. PC ranks fourth in total incidence by 7.4%, but in males, it is second only to lung cancer^4^.

Nonsurgical treatment of PC includes active surveillance (AS), androgen deprivation therapy (ADT), radiotherapy (RT), ablative therapy, chemotherapy, and emerging immunotherapy. AS is a therapeutic observation through regular monitoring of serum PSA, DRE results, serial biopsies, and diagnostic imaging (especially prostate magnetic resonance imaging (MRI)). Patients with clinically low-risk PC and some selected patients with moderate-risk disease frequently choose AS^5^. AS programs have been shown to be safe and effective^6^ in the treatment of low-risk PC. Men with localized or metastatic high-risk PC usually receive radiotherapy, while chemotherapy^7,8^ is recommended for patients with recurrent or metastatic, castration-sensitive, or drug-resistant PC. ADT is commonly used in locally advanced or advanced PC not suitable for surgery and is also considered the primary adjuvant regimen after prostate surgery. However, despite the survival benefits, ADT is associated with significant adverse effects, mainly including^9^ sexual dysfunction, gynaecomastia, anaemia, osteoporosis, and cardiovascular disease. Alternatively, ablation therapy can be used as the primary treatment for low- and medium-risk disease or as salvage therapy for^7^ clinical local disease with radiotherapy failure.

The prognosis of PC patients receiving different treatment modalities varies greatly. Although some patients adopt active surgical treatment, many patients also receive nonsurgical treatment. However, current clinical trials focus more on the prognosis of patients treated with surgery, especially those undergoing radical prostatectomy (RP)^10–12^. There are also many investigators comparing the prognosis of^13–15^ patients undergoing surgical treatment and AS. However, few studies have evaluated the relevant factors that affect the prognosis of nonsurgically treated PC patients.

The prognosis evaluation of PC in addition to the traditional TNM stage, PSA and Gleason score (GS) is also very important. The nomogram combines various clinicopathological factors, including PSA and GS, to predict cancer-specific survival (CSS) in PC patients, and there are already some nomograms for the prognosis of PC^16,17^. As a high incidence group of PC, approximately 60% of PC currently occurs in elderly men over 65 years of age and 80% of these cases are clinically localized cancers, although most patients receive active treatment^18^. However, considering the risks of excessive treatment, including the cardiopulmonary burden of anaesthesia and surgical and postoperative complications, many patients still take AS and other nonsurgical treatment modalities. However, for elderly patients who undergo nonsurgical treatment, the prognostic factors remain to be explored. However, there have been a number of risk assessment studies on the nonsurgical treatment of prostate cancer. Examples include Choi SY, Cooperberg MR, and studies by Hu et al. on the prostate^19,20^. However, these studies are limited to ADT treatment, and there is a lack of ADT data in the SEER database, so the present study focuses more attention on nonsurgical treatment, including radiotherapy and chemotherapy. To our knowledge, no predictive model can accurately predict the prognosis of elderly nonsurgically treated prostate cancer patients. We therefore aimed to develop a nomogram for nonsurgically treated elderly PC patients who can be accurately used to predict patient CSS to help clinicians and patients make decisions while avoiding overtreatment.

## Patients and methods

### Data source and data extraction

Information on PC patients over 65 years old undergoing non-surgical treatment between 2010 and 2018 was extracted from the SEER database. The SEER database serves as a national cancer database registers data from 18 cancer medical centers, covering approximately 30% of the population. Since the data in the SEER database are publicly available and the patient information is hidden, ethical approval and patient informed consent are not required. We followed the research guideline book published in the SEER database for the study.

**Figure 1:**
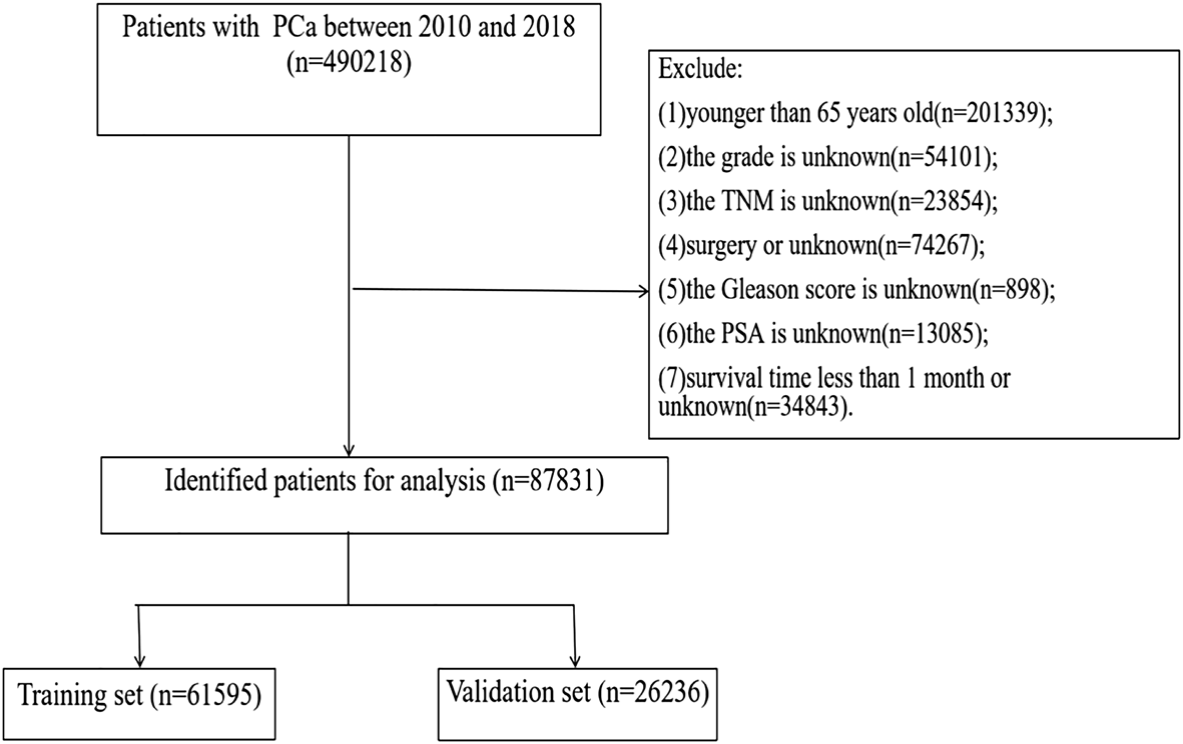
Flowchart for inclusion and exclusion of elderly non-surgical treated PC patients.

The variables we obtained from the SEER database including age, race, marital status, year of diagnosis, tumor grade, TNM stage, radiotherapy method, chemotherapy, PSA, GS, and patient follow-up outcomes, including survival status, cause of death, and survival time. The race of the patients was classified as white, black, and other types. Inclusion criteria: (1) patients aged 65 years and older; (2) a pathological diagnosis of PC; and (3) patients receiving non-surgical treatment. Exclusion criteria: (1) patients younger than 65 years old; (2) unknown tumor grade; (3) unknown TNM stage; (4) patients undergoing surgical treatment or patients with unknown surgical treatment; (5) unknown GS; (6) PSA unknown; (7) survival time less than 1 month or survival time unknown. A flowchart of patient inclusion and exclusion is shown in Fig. 1.

### Development and validation of the nomogram

All patients obtained between 2010 and 2018 were randomized to training set (70%) and validation set (30%) for nomogram development and internal validation. Independent risk factors for patients in the training set were identified using both univariate and multivariate Cox proportional regression models. A nomogram was established based on the results of the multivariate Cox regression analysis to predict CSS at 3-, 5-, and 8-years in non-surgical treated elderly PC patients. In addition, we calibrated the nomogram for 3-, 5-, and 8-years using 1000 autonomous samples. The consistency index (c-index) and the area under the subject operating characteristic curve (AUC) were used to test the accuracy and discrimination of the model.

### Clinical application

DCA is a new algorithm to calculate the net gain of the model at different thresholds. We used DCA to examine the potential clinical value of the nomogram predictive model. We also calculated the risk for each patient from the nomogram. Using the subject operating characteristic curve (ROC) as the cut-off value, all patients were divided into high-risk and low-risk groups. Differences in survival between patients at high-risk and low-risk groups were examined using the Log-rank test and Kaplan–Meier (K–M) curves. In addition, we analyzed differences of RT and chemotherapy in high-risk and low-risk groups. When analyzing the difference in chemotherapy between the high risk and low risk groups, we also performed a 1:1 propensity matching (PSM), which made the comparison cohort more reliable. The propensity-matched cohorts were counterbalanced by age, race, marriage, year of diagnosis, tumor grade, TNM stage, RT, PSA, and GS.

### Statistical analysis

Mean and standard deviation were used to describe continuous variables (age), and frequency (%) was used to describe other categorical variables (race, marriage, tumor grade, TNM stage, PSA, GS, RT and chemotherapy).Differences between groups were compared using chi-square or non-parametric U-test. The patient prognostic factors were analyzed by the Cox regression model, and the patient survival differences were analyzed by the log-rank test and the K–M curve. Statistical analyses were performed using R software version 4.1.0 and SPSS26.0. The R package includes "survival", "matching", "ggDCA", "DynNom", and "RMS". P values less than 0.05 were considered statistically significant.

### Informed consent

This study is accordance with relevant guidelines and regulations. All the data in our study were obtained from the SEER database. This is a publicly open database and does not require informed consent from the subjects and/or their legal guardians.

## Result

### Clinical features

A total of 87,831 PC patients information between 2010 and 2018 was obtained from the SEER database, all patients received non-surgical treatment and were over 65 years of age. Patients were randomized into the training set (N = 61,595) and the validation set (N = 26,236). The mean age of all patients was 72.4 ± 5.69 years, with 77.6% white and 63.3% married. The tumor grade was mainly by grade II (39.8%) and grade III (41.3%). Most patients were T1 and T2, accounting for 64.2% and 30.5%, respectively. All patients were mainly N0 (96.6%) and M0 (94.5%). 98.8% of patients did not receive chemotherapy. RT included beam radiation (42.8%), radioactive implants or isotopes (7.23%), combined with radiation (5.43%), and no radiation (44.5%). The GS were divided into GS ≤ 6 (33.5%), 7 (39.8%), and GS ≥ 8 (26.7%), respectively. Patient PSA was < 10 ng/ml (63.3%), PSA 10–20 ng/ml (20.8%), and PSA > 20 ng/ml (15.9%).There was no significant statistical bias in the clinical characteristics of both groups, and the results are shown in Table 1.

**Table 1:** Clinicopathological characteristics of elderly non-surgical treated PC patients.

	All	Training cohort	Validation cohort	P
	N = 44,975	N = 31,705	N = 13,270	
Age	72.4 (5.69)	72.4 (5.69)	72.4 (5.70)	0.646
Race				0.762
White	68,190 (77.6%)	47,781 (77.7%)	20,409 (77.5%)	
Black	11,788 (13.4%)	8254 (13.4%)	3534 (13.4%)	
Other	7853 (8.94%)	5471 (8.90%)	2382 (9.05%)	
Marital				0.623
No/unknown	32,252 (36.7%)	22,618 (36.8%)	9634 (36.6%)	
Married	55,579 (63.3%)	38,888 (63.2%)	16,691 (63.4%)	
Grade				0.882
I	16,464 (18.7%)	11,569 (18.8%)	4895 (18.6%)	
II	34,919 (39.8%)	24,428 (39.7%)	10,491 (39.9%)	
III	36,284 (41.3%)	25,396 (41.3%)	10,888 (41.4%)	
IV	164 (0.19%)	113 (0.18%)	51 (0.19%)	
T				0.308
T1	56,348 (64.2%)	39,576 (64.3%)	16,772 (63.7%)	
T2	26,788 (30.5%)	18,662 (30.3%)	8126 (30.9%)	
T3	3701 (4.21%)	2584 (4.20%)	1117 (4.24%)	
T4	994 (1.13%)	684 (1.11%)	310 (1.18%)	
N				0.279
N0	84,841 (96.6%)	59,385 (96.6%)	25,456 (96.7%)	
N1	2990 (3.40%)	2121 (3.45%)	869 (3.30%)	
M				0.298
M0	83,022 (94.5%)	58,171 (94.6%)	24,851 (94.4%)	
M1	4809 (5.48%)	3335 (5.42%)	1474 (5.60%)	
Chemotherapy				0.086
No	86,784 (98.8%)	60,747 (98.8%)	26,037 (98.9%)	
Yes	1047 (1.19%)	759 (1.23%)	288 (1.09%)	
Radiation				0.350
No	39,118 (44.5%)	27,279 (44.4%)	11,839 (45.0%)	
Beam radiation	37,597 (42.8%)	26,391 (42.9%)	11,206 (42.6%)	
Radioactive implants or isotopes	6347 (7.23%)	4479 (7.28%)	1868 (7.10%)	
Combination	4769 (5.43%)	3357 (5.46%)	1412 (5.36%)	
Gleason				0.331
≤ 6	29,393 (33.5%)	20,668 (33.6%)	8725 (33.1%)	
7	34,944 (39.8%)	24,383 (39.6%)	10,561 (40.1%)	
≥ 8	23,494 (26.7%)	16,455 (26.8%)	7039 (26.7%)	
PSA				0.339
< 10	55,621 (63.3%)	38,993 (63.4%)	16,628 (63.2%)	
10–20	18,247 (20.8%)	12,700 (20.6%)	5547 (21.1%)	
> 20	13,963 (15.9%)	9813 (16.0%)	4150 (15.8%)	
CSS				0.950
Dead	4442 (5.06%)	3113 (5.06%)	1329 (5.05%)	
Alive	83,389 (94.9%)	58,393 (94.9%)	24,996 (95.0%)	
Survival months	47.0 (29.0)	47.0 (29.0)	47.2 (29.0)	0.211

### Univariate and multivariate COX regression analysis

Univariate Cox regression models were used to analyze and screen for influencing factors associated with survival in the training set.The results showed that age, race, marriage, Grade tumor grade, TNM stage, chemotherapy, RT, PSA, and GS were all prognostic factors affecting patient survival.Then, independent risk factors associated with patient survival were screened by multivariate Cox regression analysis. Results found that age, race, marriage, TNM stage, RT method, chemotherapy, PSA and GS were independent risk factors for CSS, and tumor stage was not an independent risk factor for CSS.All of the results are shown in Table 2.

**Table 2:** Univariate and multivariate analyses of CSS in training cohort.

	Univariate			Multivariate		
	HR	95% CI	P	HR	95% CI	P
Age	1.1	1.1–1.11	< 0.001	1.064	1.061–1.067	< 0.001
Race						
White						
Black	1.22	1.11–1.35	< 0.001	1.244	1.186–1.305	< 0.001
Other	0.61	0.53–0.71	< 0.001	0.583	0.544–0.625	< 0.001
Marital						
No						
Married	0.77	0.72–0.83	< 0.001	0.817	0.789–0.845	< 0.001
Grade						
I						
II	2.14	1.62–2.82	< 0.001			
III	13.56	10.44–17.6	< 0.001			
IV	23.82	12.5–45.4	< 0.001			
T						
T1						
T2	2.12	1.96–2.3	< 0.001	1.146	1.105–1.189	< 0.001
T3	5.46	4.85–6.15	< 0.001	1.124	1.045–1.209	0.002
T4	23.2	20.36–26.44	< 0.001	1.737	1.578–1.911	< 0.001
N						
N0						
N1	11.25	10.29–12.3	< 0.001	1.271	1.187–1.362	< 0.001
M						
M0						
M1	26.44	24.59–28.44	< 0.001	2.742	2.593–2.9	< 0.001
Chemotherapy						
No/unknown						
Yes	10.5	9.12–12.09	< 0.001	1.202	1.079–1.34	0.001
Radiation						
No/unknown						
Beam radiation	0.42	0.39–0.45	< 0.001	0.694	0.667–0.721	< 0.001
Radioactive implants or isotopes	0.12	0.09–0.16	< 0.001	0.637	0.586–0.692	< 0.001
Combination	0.25	0.2–0.32	< 0.001	0.513	0.466–0.565	< 0.001
PSA						
< 10						
10–20	2.44	2.18–2.72	< 0.001	1.273	1.217–1.331	< 0.001
> 20	12.37	11.36–13.46	< 0.001	1.62	1.545–1.699	< 0.001
Gleason						
≤ 6						
7	2.57	2.21–3	< 0.001	1.45	1.38–1.524	< 0.001
≥ 8	17.2	15.01–19.72	< 0.001	2.204	2.088–2.327	< 0.001

### Development and validation of the nomograms

**Figure 2:**
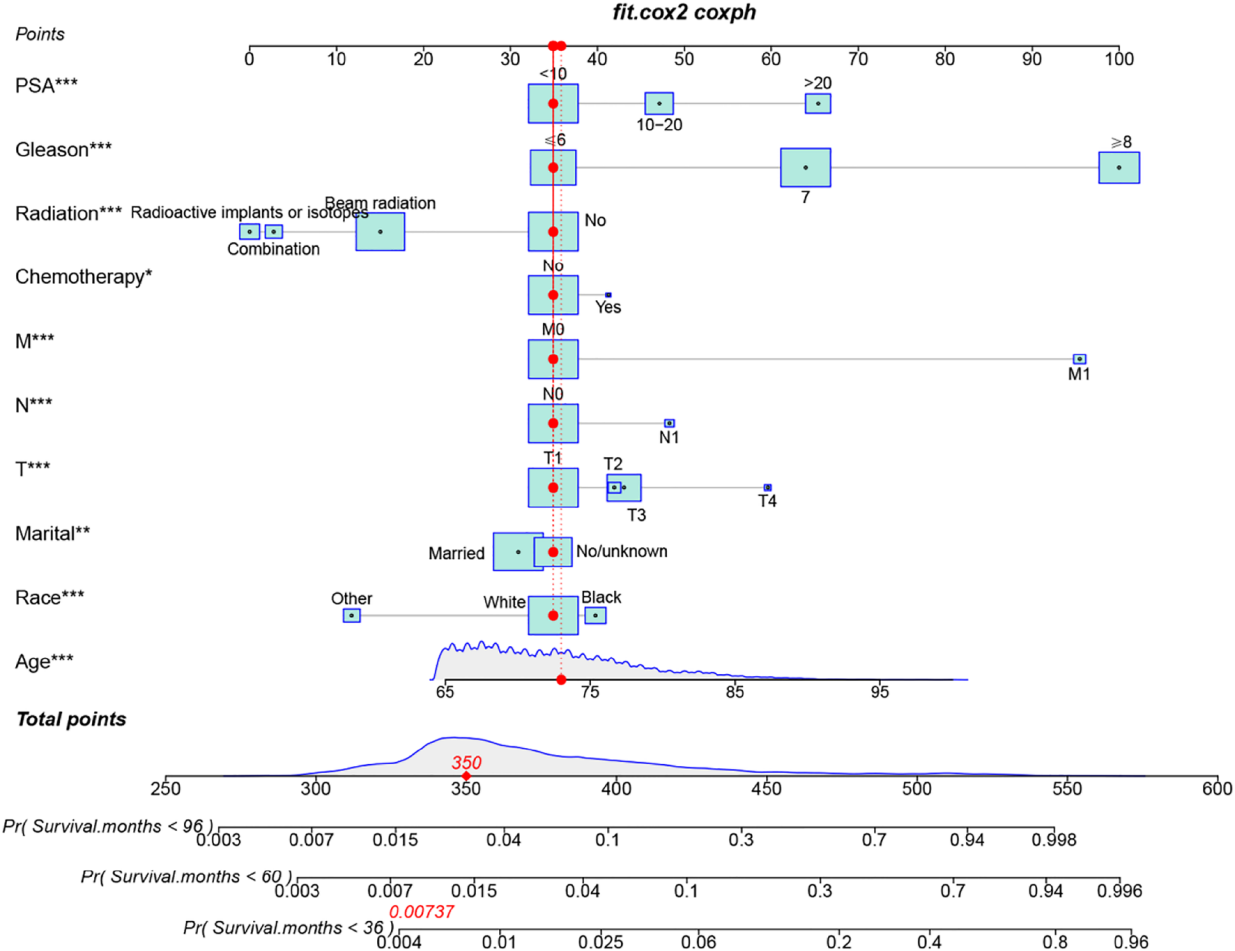
The nomogram for predicting 3-, 5-, 8-year CSS in elderly PC patients undergoingelderly non-surgical treated PC patients.

We constructed a new nomogram based on the multivariate Cox regression analysis model to predict CSS at 3, 5, and 8 years in non-surgical treated elderly PC patients (Fig. 2). The nomogram showed that PSA, GS, radiotherapy mode and TNM stage were the most critical factors affecting CSS in elderly PC treated patients. In addition, age, marriage, race, and chemotherapy also had some influence on CSS.

**Figure 3:**
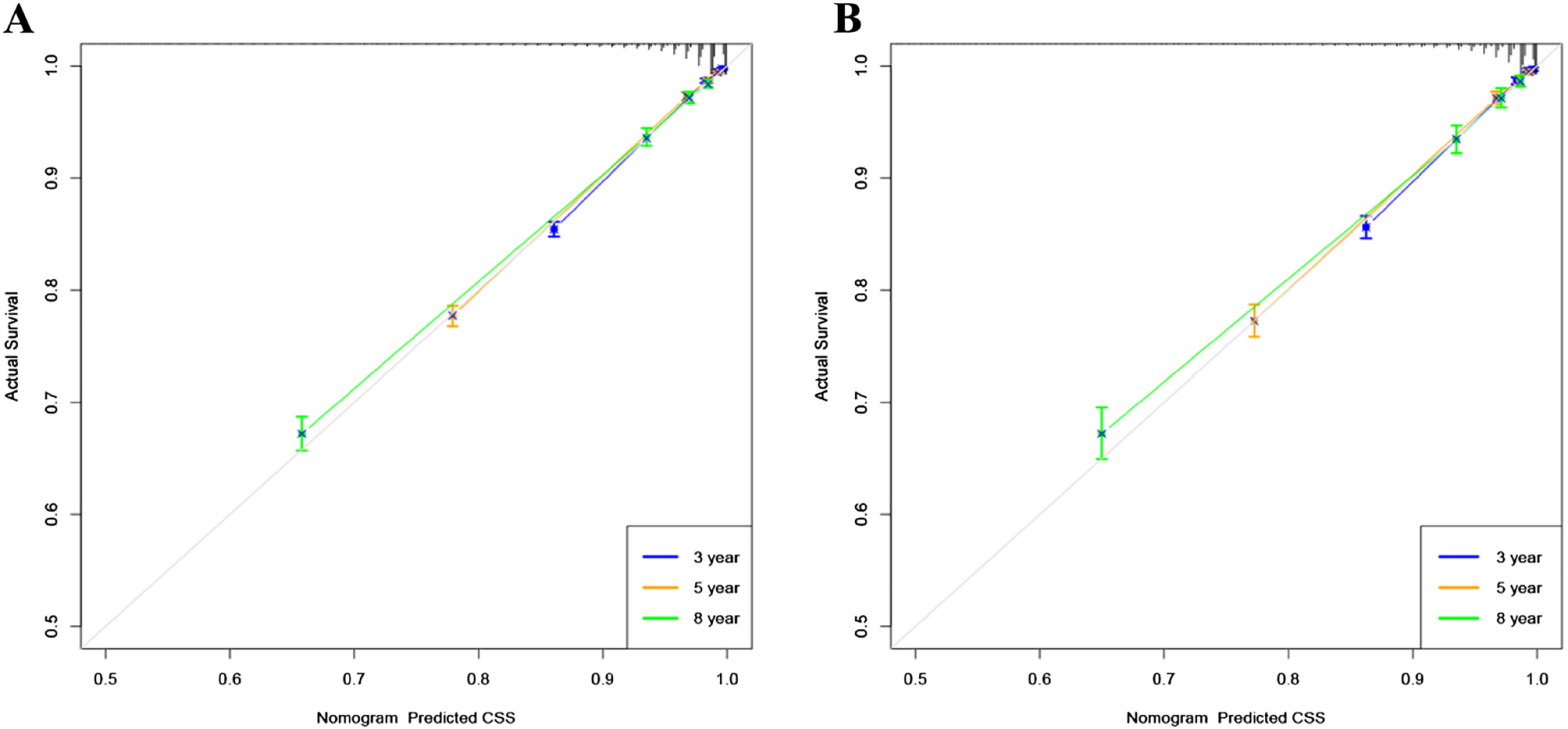
Calibration curve of the nomogram in the training set (**A**) and validation set (**B**). The horizontal axis is the predicted value in the nomogram, and the vertical axis is the observed value.

**Figure 4:**
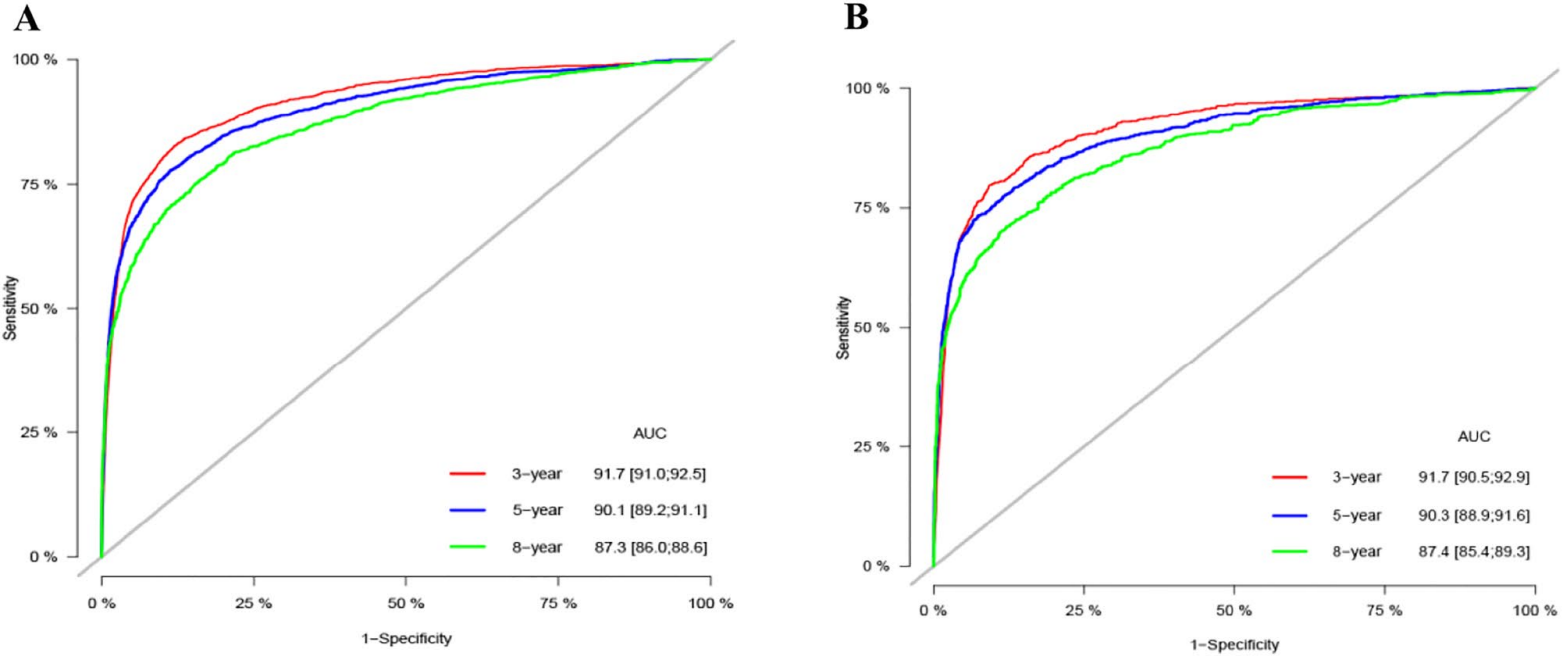
AUC for predicting 3-, 5-, and 8-year CSS in the training set (**A**) and validation set (**B**).

Internal cross-validation was used to verify the accuracy and discriminability of the model. The C-index for the training and validation set is 0.894 (95% CI 0.888–0.900) and 0.897 (95% CI 0.887–0.907), respectively, indicating that the prediction model has good recognition ability. The calibration curve indicates that the nomogram has good accuracy, and the results show that the predicted values of the nomogram are highly consistent with the actual observed values (Fig. 3). In the training set, the AUC at 3-, 5-, and 8-years was 91.7, 90.1, and 87.3, respectively, and in the validation set, the AUC was 91.7, 90.3, and 87.4 at 3-, 5-, and 8-years (Fig. 4), showing good discrimination of the nomogram.

### Clinical application of the nomogram

**Figure 5:**
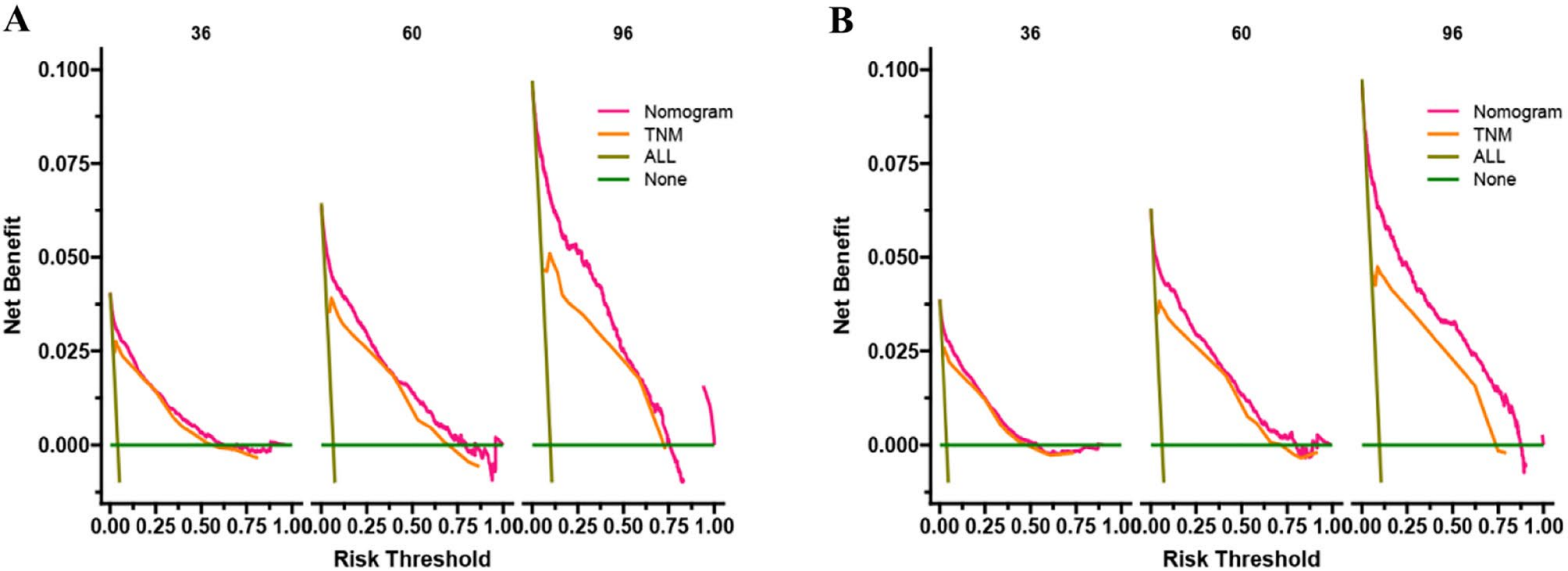
DCA of the nomogram in the training set (**A**) and the validation set (**B**). The Y-axis represents a net benefit, and the X-axis represents threshold probability. The green line means no patients died, and the dark green line means all patients died. When the threshold probability is between 0 and 100%, the net benefit of the model exceeds all deaths or none.

**Figure 6:**
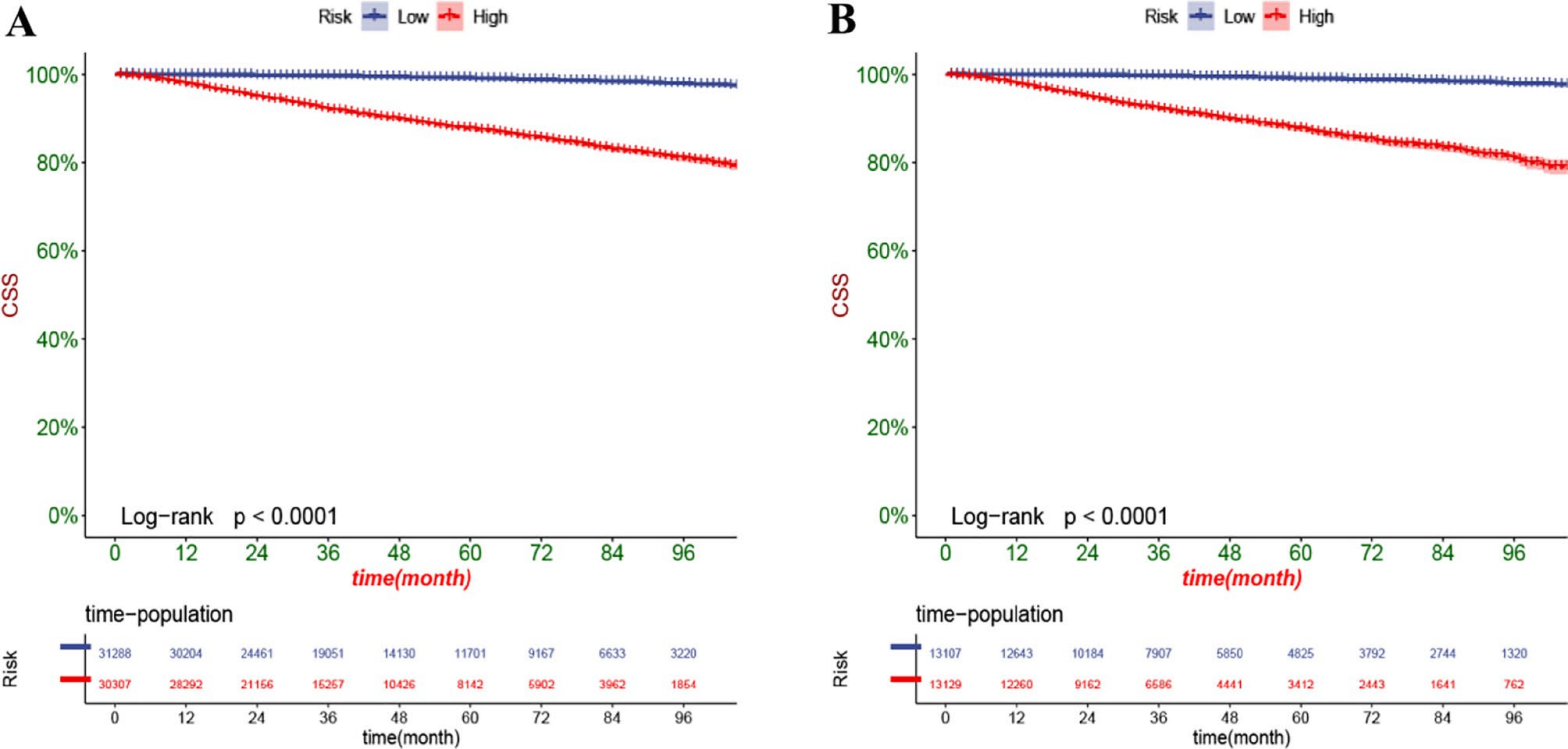
Kaplan–Meier curves of patients in the low-risk and high-risk groups in the training set (**A**) and validation set (**B**).

**Figure 7:**
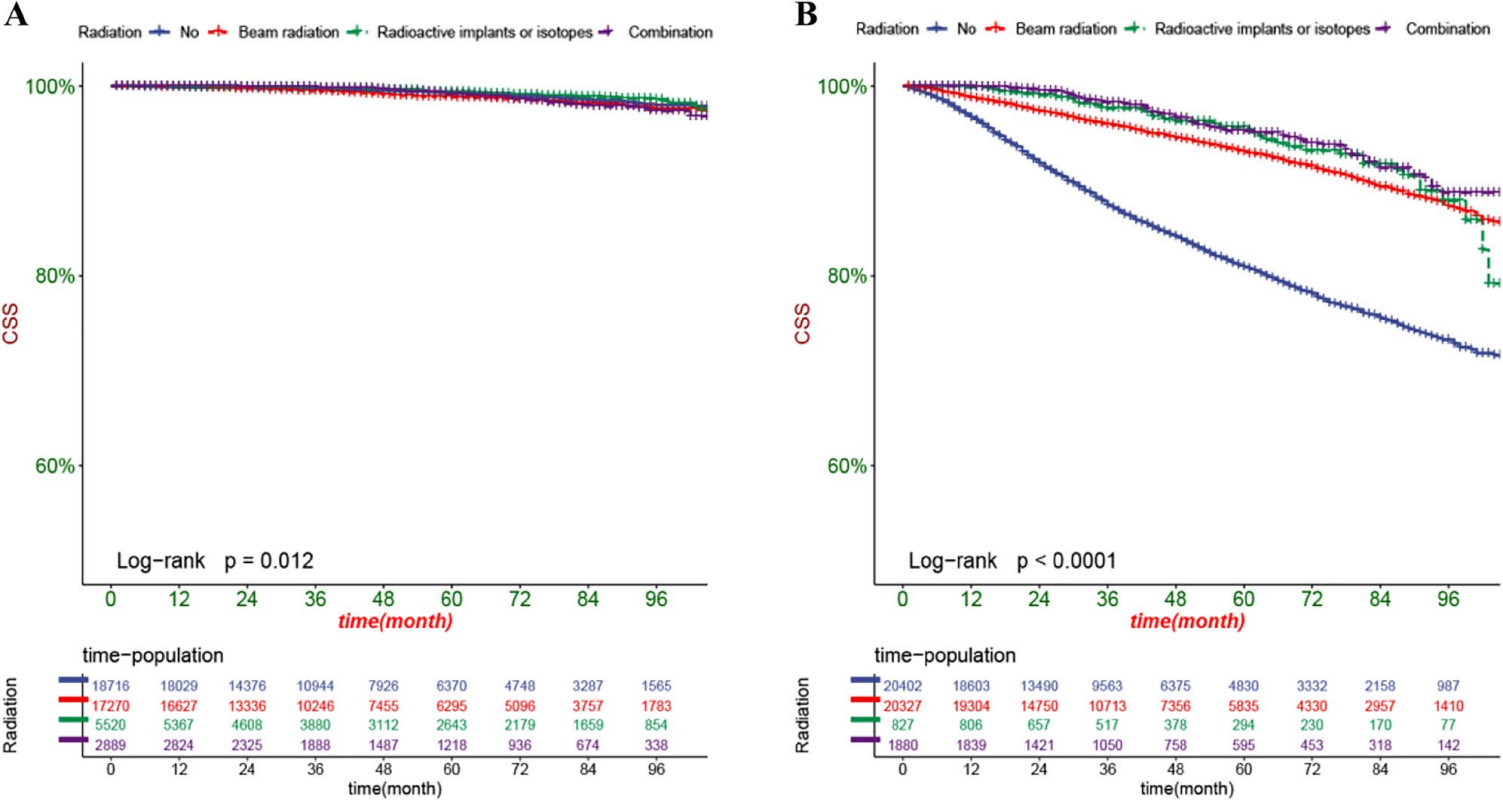
Kaplan–Meier curves of patients with different radiotherapy in the low-risk group (**A**) and high-risk group (**B**).

**Figure 8:**
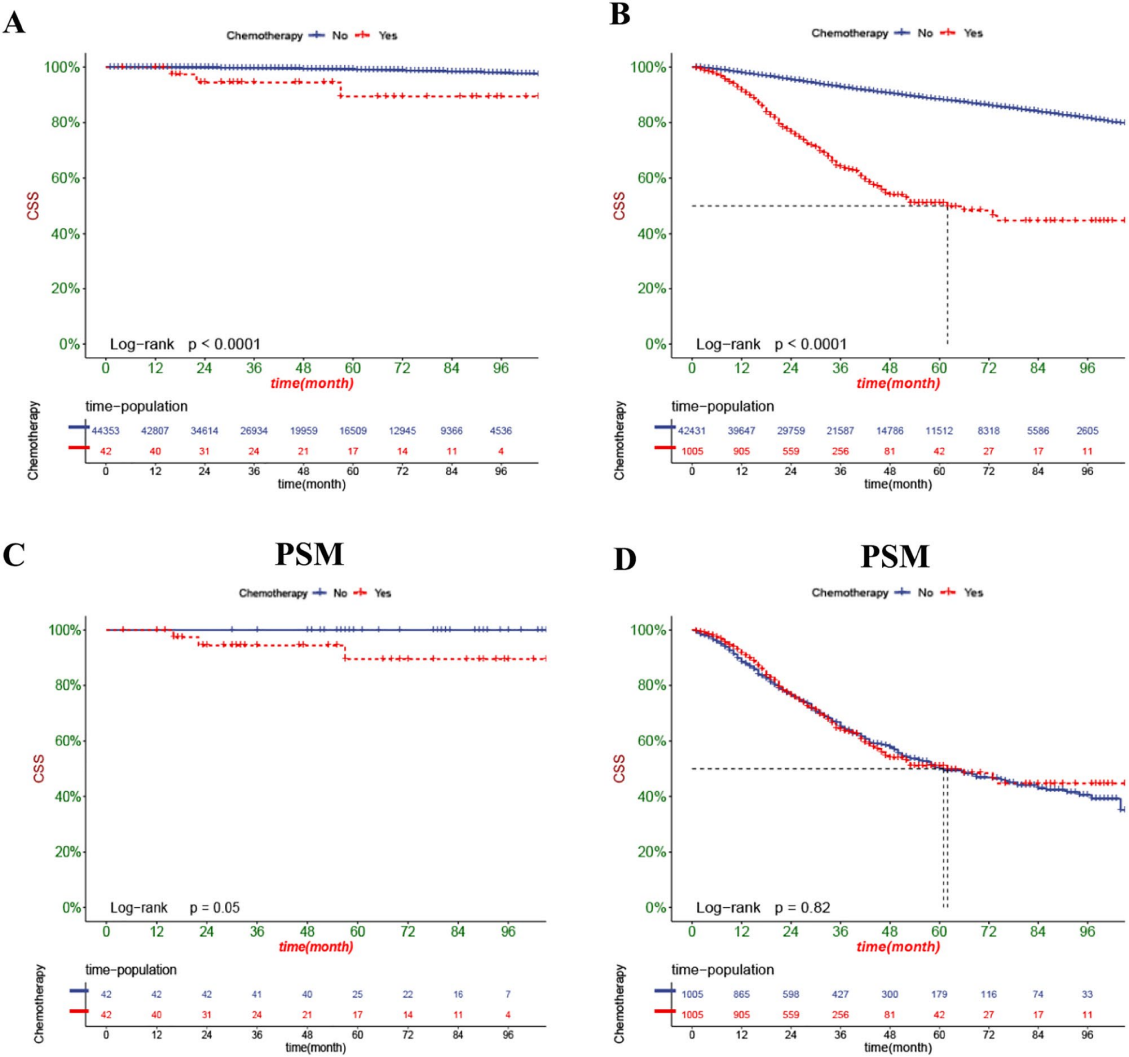
Kaplan–Meier curves of patients with or without chemotherapy in the low-risk group (**A**) and high-risk group (**B**). Kaplan–Meier curves after PSM of patients with or without chemotherapy in the low-risk group (**C**) and high-risk group (**D**).

The DCA can assess the potential clinical value of the nomogram, and the results show a good clinical potential value in both the training group and the validation set (Fig. 5). Furthermore, we calculated the risk values for each patient based on the nomogram, and the ROC curve was used to calculate the optimal cut-off value. Patients were divided by cut-off into high risk (total score > 153.78) and low risk (total score < 153.78).The K–M curves of both the training and validation sets showed that the survival rate of the patients in the low-risk group was significantly higher than that in the high-risk group (Fig. 6). In the high-risk group, the 3-, 5-, and 8-year CSS were 92.2%, 87.7%, and 81.0%, respectively.In the low-risk group, the 3-year, 5-year, and 8-year CSS were 99.6%, 99.1%, and 97.9%, respectively.The K–M curve analysis of RT showed that among the patients in the high-risk group, patients treated with radioactive implants or isotope and combined with radiotherapy had the best prognosis, and there was no significant survival difference between the two radiotherapy methods, followed by patients receiving beam radiation, while patients without radiotherapy had the worst prognosis; and there was no significant difference in survival between any of the four RT modalities in the low-risk group (Fig. 7). We also conducted a K–M curve analysis of chemotherapy methods, and found that the survival rate of the high-risk and low-risk groups was lower than those without chemotherapy, and too few people considered chemotherapy, resulting in outcome bias.Therefore, we matched patients 1:1 and further analyzed the survival differences of chemotherapy. The results showed that after PSM, in the high-risk group, there was no significant difference between the survival rate of chemotherapy and no chemotherapy, while in the low-risk group, the survival rate of patients receiving chemotherapy was still lower, considering the reason that most low-risk patients can achieve better treatment effect without chemotherapy, and the side effects of chemotherapy lead to worse prognosis (Fig. 8).

## Discussion

PC is the most common noncutaneous malignancy in men in the United States and its incidence is only increasing, with 268,490 new patients expected in the United States by 2022 and an expected 34,500 deaths from PC in the United States^21^. Worldwide, PC accounts for 37% of malignant solid tumours in males, and an estimated 19.3 million new cancer cases were reported worldwide in 2020. Treatment modalities for PC patients include surgical treatment and nonsurgical treatment. With the popularity of serum PSA screening, the proportion of early PC diagnoses has gradually increased, and the median age at diagnosis of PC has gradually advanced from 72 years old to 66 years old^22^. The proportion of nonsurgical treatment is also increasing^1^. PC has a high incidence in the elderly population, so this study used the SEER database to establish a new nomogram to predict CSS in nonsurgically treated elderly PC patients, and our results showed that age, race, marriage, PSA, GS, RT mode, chemotherapy and TNM stage are independent risk factors for elderly prostate patients 3 and 8 years.

With PC as the most common malignancy in developed countries, most patients have an age of diagnosis of over 65 years of age^23^. Regarding the effect of age on malignant potential, several malignancies show different characteristics between older and younger patients. In gastric cancer as well as colon cancer, younger patients have a worse prognosis than older patients^24,25^. However, a meta-analysis showed a 5-year OS of 62.1% in PC patients aged 56–65 years old, compared with 59.8% aged 66–75 years old, indicating that elderly PC patients have a worse prognosis^26^. Our study also showed that age is an important factor in CSS in PC.

Previous literature has reported that the risk of PC varies between ethnic groups, with increased risk in African men and reduced^27^ risk in Asian men. PC risk factors in Asian men may differ significantly from those in white men, and subtypes may also vary in^28^ by ethnicity. Our predictive model suggests that race in aged nonsurgically treated PC patients is also an independent risk factor, and nomograms indicate that Black patients have the worst prognosis, followed by White, and Asian and other ethnic groups have the best prognosis. Alternatively, marriage is a protective factor for many cancer patients, for example, breast cancer and bladder cancer^29,30^. Considering the financial support and psychological comfort of marriage, some studies have also shown that marital status is closely related to the prognosis of PC^31^, which is consistent with our results.

Our nomogram shows that TNM stage is also an independent risk factor for CSS in elderly nonsurgical PC patients, and patients with distant as well as lymph node metastases have worse outcomes than nonmetastases and higher T stage, which is consistent with previous studies^32^. It is well known that PSA is an important factor affecting patient prognosis. Our prediction model shows that the level of PSA is also an independent risk factor affecting the CSS of PC. The results show that most elderly nonsurgical PC patients have low PSA levels, mostly below 10, and patients with PSA < 10 have a much higher prognosis than those with PSA 10–20 and above 20. In 1974, Gleason and Mellinger proposed the GS scoring system, where GS remains the most powerful predictor of PC prognosis^33^ compared to other clinicopathological factors and molecular markers. Although different GS groups were used in clinical practice, most researchers grouped GS by GS ≤ 6, GS7, and GS ≥ 8, and most studies showed higher GS and worse patient prognosis^34–36^. Our results showed that most GS of elderly nonsurgically treated PC patients were GS ≤ 6, which is consistent with previous literature.

RT provides a treatment for localized PC without major surgery and is the preferred treatment in many men^37^. Approximately 30% of PC patients receive RT each year^38^. PC patients with adverse pathological features after prostatectomy may benefit from postoperative RT^39^. RT for PC mainly includes external beam radiotherapy (EBRT), in which radiation beams produced by in vitro machines are exposed to cancer cells, brachytherapy, where radioactive substances and other isotopes are placed near cancer cells in the body, or EBRT and brachytherapy combined^40^. PSA has been introduced as a screening tool and is now also used as a marker of response to RT. Although it has been shown that serum PSA can appear as temporary and benign "PSA rebound" in the early stages after RT, it does not necessarily indicate PC treatment failure. PSA rebound can occur after external beam or brachytherapy and occurs in 30–40% of successfully treated men^41^. A randomized trial conducted by Christopher et al. showed that RT, although it improved asymptomatic survival in unselected patients with newly diagnosed metastatic PC, did not improve overall survival. However, for patients with a low metastatic burden, RT improved 3-year overall survival and failure-free survival^42^. The results of a phase 3 randomized trial conducted by Linda et al. showed that external irradiation radiation does not improve the CSS and OS of PC^43^. However, this conclusion is controversial. Freddie et al. showed that by targeting localized PC, RT significantly reduced disease progression and metastasis incidence^44^. The randomized trial conducted by Lars Holmberg et al. showed that RT significantly reduced mortality in PC^45^. Our findings suggest that patients who receive RT have better outcomes than those who do not. Moreover, we also found that among the patients receiving RT, the patients receiving external beam irradiation had the worst prognosis, while the internal implantation and combination treatment were better, with no significant difference between the two groups.

Since the 1940s, androgen ablation therapy has been the mainstay of treatment for prostate cancer, but drug resistance has developed over time. Thus, between the 1950s and 1970s, a number of small trials using alkylating agents were conducted, which was the original chemotherapy for prostate cancer. However, the initial chemotherapy results were not significant. In 1972, the National Prostate Cancer Project (NPCP) began a series of randomized single-agent and phase III combination studies called "hormone resistance" but later created "castration-resistant" PCa patients. Chemotherapy is not the primary treatment for prostate cancer, but docetaxel has improved overall survival in metastatic castration-resistant prostate cancer (mCRPC); however, combination chemotherapy or any drug added to docetaxel has failed to produce more benefit^46^. The recent proposal of the combination of ADT + androgen receptor-targeted agents (ARTA) or ADT + ARTA + docetaxel for mHSPC patients overcomes this clinical situation. B. A. Maiorano et al. also supported the combination of ARTA with docetaxel and ADT in patients with mHSPC^47^. Our nomogram showed that chemotherapy is also an independent risk factor for prognosis, and our K‒M curve analysis revealed that survival was lower in high-risk and low-risk groups than in patients without chemotherapy, and we considered too few people receiving chemotherapy to cause outcome bias. Therefore, we further analysed the survival difference of chemotherapy methods after the 1:1 PSM of patients. The results showed that after PSM, there was no significant difference between chemotherapy and without chemotherapy in the high-risk group, while in the low-risk group, the CSS of patients receiving chemotherapy was still lower, possibly because most low-risk patients can obtain better treatment effects without chemotherapy, and the side effects of chemotherapy lead to worse prognosis.

Although the nomogram established based on the SEER database has good accuracy, there are some potential limitations simultaneously, including the lack of some important clinicopathological variables, such as smoking, alcohol consumption, and haemoglobin. In addition, AS and ADT are also the main nonsurgical treatments for nonsurgical PC patients. Meanwhile, ADT, as one of the nonsurgical treatment options for patients with prostate cancer, is usually used for high-risk local or systemic advanced PC that is not suitable for radical surgery. Although ADT was explored in Beebe-Dimmer and Muralidhar et al. in the SEER-based study^48,49^. However, their ADT data are all from the SEER-Medicare database, and the SEER-Medicare database database is only open to Americans. We cannot obtain relevant access rights, so we cannot obtain relevant data for research. However, the SEER database lacks data related to AS and ADT, so our model also lacks the relationship between AS and ADT and prognosis. Furthermore, database-based studies are all retrospective, which may confer a risk of unavoidable selection bias. Future prospective studies with large and multicentre samples are needed for further validation of the nomogram. Finally, although our nomogram could not include all prognostic-related variables, such as BMI, smoking, and drinking, we still included most of the key clinical factors and conducted internal verification, so there would not be a large deviation in the results.

## Conclusion

Our study developed a predictive model for nonsurgically treated elderly PC patients with all data from the SEER database, and we found that age, race, marriage, TNM stage, PSA, GS, RT modality, and chemotherapy were independent risk factors affecting patient CSS. The model has been internally validated with good accuracy and reliability and can be used for adjuvant decision-making in elderly prostate cancer patients.

## Data Availability

The SEER data analyzed in this study is available at https://seer.Cancer.gov/.
